# Comparison of heavy metal contents, fatty acid composition, and palinological characteristics of two different *Hypericum* species growing in mining-impacted area

**DOI:** 10.3389/fpls.2026.1900502

**Published:** 2026-08-27

**Authors:** Mihriban Ahıskalı

**Affiliations:** Bingöl University Vocational School of Food, Agriculture and Livestock, Bingöl, Türkiye

**Keywords:** fatty acid, heavy metal, *Hypericum*, mining area, palinology

## Abstract

**Introduction:**

This study comparatively evaluated heavy metal (Al, Cd, Cr, Fe, and Mn) concentrations, fatty acid composition, and palynological characteristics in *Hypericum scabrum* and *Hypericum lydium* samples collected from mining-impacted and reference sites of an iron ore operation.

**Methods:**

Plant samples of *H. scabrum* and *H. lydium* were collected from mining-impacted and reference sites in Bingöl Province, Türkiye. Heavy metal concentrations, fatty acid composition, and pollen morphological characteristics were analyzed using ICP-MS, gas chromatography, and light microscopy, respectively. Translocation factor (TF) and statistical analyses were also performed.

**Results:**

In both species, Al, Cr, Fe, and Mn accumulation was markedly increased in the mining area, whereas the increase in Cd remained relatively limited with statistically significant differences between sites. Translocation factor (TF) analyses revealed higher values for most metals in the mining area; notably, *H. lydium* exhibited a greater translocation capacity for Fe and Mn. In contrast, TF values for Cr remained below 1 in both species, indicating its retention at the root level. Mining conditions were found to markedly alter the fatty acid composition in both Hypericum species, particularly affecting the balance between saturated and unsaturated fatty acids. Under heavy metal stress, total saturated fatty acids (ΣTSFA; particularly palmitic acid (C16:0) and stearic acid (C18:0)) increased in the mining area, whereas total unsaturated fatty acids (ΣTUSFA; oleic acid (C18:1), linoleic acid (C18:2), and α-linolenic acid (C18:3)) were higher under non-mining conditions. From a palynological perspective, the increased metal load preserved morphological integrity in the pollen of *H. scabrum*, whereas it caused pronounced changes in the P/E ratio and pollen shape in *H. lydium*, with statistically significant differences observed.

**Discussion:**

Overall, the findings suggest that mining-related heavy metal stress influences metal accumulation, fatty acid composition, and pollen characteristics in *Hypericum* species, although the magnitude and nature of these effects differ between *H. scabrum* and *H. lydium*. These species-specific differences indicate that the two species exhibit distinct physiological and morphological responses to heavy metal stress, contributing to a better understanding of plant–heavy metal interactions in mining ecosystems.

## 1 Introduction

Heavy metal pollution, particularly originating from mining activities, represents one of the most significant and increasingly prevalent environmental stress factors affecting soil ecosystems. In high-extraction areas such as iron ore mining sites, the accumulation of elements like Al, Cd, Cr, Fe, and Mn in the soil exerts both direct and indirect effects on the physiological and biochemical processes of plants (Sharma and Dubey, 2005; DalCorso et al., 2008). Numerous studies have reported that these metals significantly affect the vegetative organs of plants. Moreover, the impacts of heavy metal stress are not limited to vegetative tissues but also extend to generative processes (Zheljazkov and Nielsen, 1996). Pollen grains serve as an important bio-indicator for revealing the generative-level effects of heavy metal stress, reflecting anthropogenic environmental pollution (Dinkov and Stratev, 2016). The literature reports that Cd, Pb, and other heavy metals reduce pollen viability, cause structural disruptions in the anther tissue, and induce changes in pollen morphometric parameters (Mohsenzadeh et al., 2011; Yousefi et al., 2011). Changes in exine and intine thickness, shifts in the P/E ratio, and alterations in pollen shape are considered palynological indicators of environmental stress. Elevated heavy metal concentrations in the soil, associated with environmental pollution, induce stress in plants, which can affect their physiological processes, limit productivity, and potentially lead to plant mortality (Ergün et al., 2012). Soil metal concentrations, in addition to being influenced by human activities, also depend on the geological origin of the soil and can range from 1 mg/kg (ppm) to 100,000 mg/kg (Blaylock and Huang, 2000). The permissible limits for toxic metals in soil and plants have been established by the World Health Organization (WHO). For Cd, the limits are 3 mg kg^-1^ in soil and 0.2 mg kg^-1^ in plants; for Cr, 150 mg kg^-1^ in soil and 5 mg kg^-1^ in plants; for Fe, 50–000 mg kg^-1^ in soil and 450 mg kg^-1^ in plants; and for Mn, 80 mg kg^-1^ in soil and 500 mg kg^-1^ in plants (WHO/FAO, 2007; Sönmez and Kılıç, 2021). Plants grown in uncontaminated areas contain lower levels of heavy metals compared to those from polluted sites. Humans may consume plants collected from nature, particularly those with medicinal properties. While medicinal plants exert pharmacological effects, their safety can be compromised by the presence of heavy metals such as Pb, Ni, Cd, and Zn, which may render them toxic. Therefore, the contaminant levels in plants intended for medicinal use should be carefully monitored (Baranowska et al., 2002). The genus *Hypericum* L. (Hypericaceae) is widely distributed worldwide and includes species of significant ecological and pharmacological importance. Due to their secondary metabolite content and tolerance to environmental stresses, *Hypericum* species have been the subject of numerous ecophysiological studies (Ayan et al., 2006). However, comprehensive studies simultaneously evaluating heavy metal accumulation, fatty acid composition, and palynological parameters in *Hypericum* species are limited. Extreme habitats, such as mining sites, where heavy metals and other abiotic stress factors are prevalent, provide a natural research setting to investigate the physiological, biochemical, and morphological adaptation mechanisms that plants develop in response to environmental stress under natural conditions.

Recent studies have shown that heavy metal stress induces a wide range of physiological and biochemical responses in plants, including alterations in metal uptake, transport, detoxification processes, and antioxidant defense mechanisms (Raza et al., 2021; Al Obaidi et al., 2024). The excessive accumulation of heavy metals can enhance reactive oxygen species (ROS) production, resulting in oxidative stress and disturbances in cellular structures, particularly membrane systems (Raza et al., 2021). Since membrane lipids are highly sensitive to oxidative damage, changes in lipid metabolism and fatty acid composition are considered important components of plant responses to heavy metal stress. Modifications in fatty acid unsaturation levels may contribute to maintaining membrane stability and cellular function under unfavorable environmental conditions (He and Ding, 2020). In addition, pollen characteristics have been increasingly used in environmental stress studies, as alterations in pollen size, shape, viability, and wall structures, including exine features, may reflect the effects of environmental pollution on plant reproductive processes (Vasilevskaya, 2022). However, previous studies have generally focused on heavy metal accumulation, biochemical responses, or reproductive traits separately. To the best of our knowledge, few studies have simultaneously evaluated heavy metal accumulation, fatty acid composition, and palynological characteristics in *Hypericum* species growing under contrasting habitat conditions. Therefore, an integrated assessment of elemental accumulation, biochemical responses, and reproductive traits is necessary to better understand species-specific responses to heavy metal stress in mining ecosystems.

This study aimed to comparatively assess heavy metal concentrations and fatty acid compositions in two widely used medicinal *Hypericum* species (*H. scabrum* and *H. lydium*) collected from different habitat conditions (mining-impacted and reference sites). Accordingly, Al, Cd, Cr, Fe, and Mn contents were analyzed in individuals from both habitats, while saturated and unsaturated fatty acid components were simultaneously evaluated. Additionally, the potential effects of heavy metal accumulation on pollen morphology were investigated, and the possible reflections of habitat-related environmental differences on palynological traits were examined. To date, no comprehensive study has holistically addressed heavy metal accumulation, fatty acid profiles, and palynological characteristics of any *Hypericum* species within the same research framework and based on habitat comparisons.

Accordingly, the study examined the variations in heavy metal contents and saturated/unsaturated fatty acid components in roots, stems, leaves, and flowers under mining-impacted and reference site conditions. In addition, it evaluated whether these habitat differences produced measurable effects on palynological traits. This approach enables a holistic analysis of the biochemical and morphological responses of the species to ecological conditions.

## 2 Materials and methods

### 2.1 Study area

Plant materials used in this study were collected between April and June 2024 from two distinct locations within Bingöl Province. The first site was the Avnik Iron Mine, located approximately 40 km from the city center of Bingöl, which has been actively operating for around 15 years. This site was selected due to its elevated soil heavy metal concentrations compared to the reference area. The second site, serving as a reference (control) area free from mining influence, was a forested region within the Bingöl University Campus. The reference site was selected to represent natural environmental conditions without direct anthropogenic mining disturbance and was used as a baseline for evaluating the potential effects of mining activities on heavy metal accumulation, fatty acid composition, and palynological characteristics of the studied *Hypericum* species. This site was selected because previous studies reported elevated soil heavy metal concentrations in the mining-impacted area compared with uncontaminated locations. Although the mining-impacted and reference sites are located within the same region and have similar climatic conditions, including precipitation patterns, the presence of mining activities has resulted in differences in soil characteristics, particularly in terms of heavy metal concentrations. Therefore, the reference site provides an appropriate comparison area for assessing the effects of mining-related environmental changes on the investigated plant species. Her The physicochemical properties of soils from the two sampling sites are presented in **Table 1**. For each sampling location, coordinates and elevation were recorded, and soil pH, electrical conductivity (EC, µS cm^−1^), calcium carbonate content (CaCO_3_, %), organic matter content (%), and total element concentrations (mg kg^−1^) were determined. Soil metal data were obtained from previously published studies for the same area and used for comparative interpretation.

**Table 1:** Position and characteristics of the study areas.

	Non-mining site (Gökdere et al., 2026)	Mining site (Şahin and İnci, 2026b)
pH	6.88	6.60
EC (µs)	190.30	403.10
CaCO3 (%)	1.96	2.58
Organic matter (%)	0.38	0.41
Total Element Concentrations (mg kg^-1^)	K:354.50, P:21.23 Cd:0.06, Si: 7.60, Zn:1.71, Fe:24.02, Mn:32.74, Ca:7322.01, Mg:301.22	Al:21832,80, Co:6.50, Cr:1.50 Cu:1.70, Fe:9821.50, Mn:117.20, Ni:7.60, Pb:2.30, Zn:5.20
Altitude	1163 m	1240 m
Coordinates	38°53’ N 40°29’ E,	38° 39’ 1”N- 40° 18’ 13” E

Soil metal data were obtained from previously published studies for the same area and used for comparative interpretation. Previous investigations have demonstrated that soils surrounding the Avnik iron mine differ from reference soils in terms of elemental composition, mainly due to the geological characteristics of the region and mining activities. The mining-impacted soils have been reported to contain elevated levels of Fe and associated elements compared with non-mining reference areas (Taş and Demir, 2022; Kırat and Vural, 2024).

**Figure 1:**
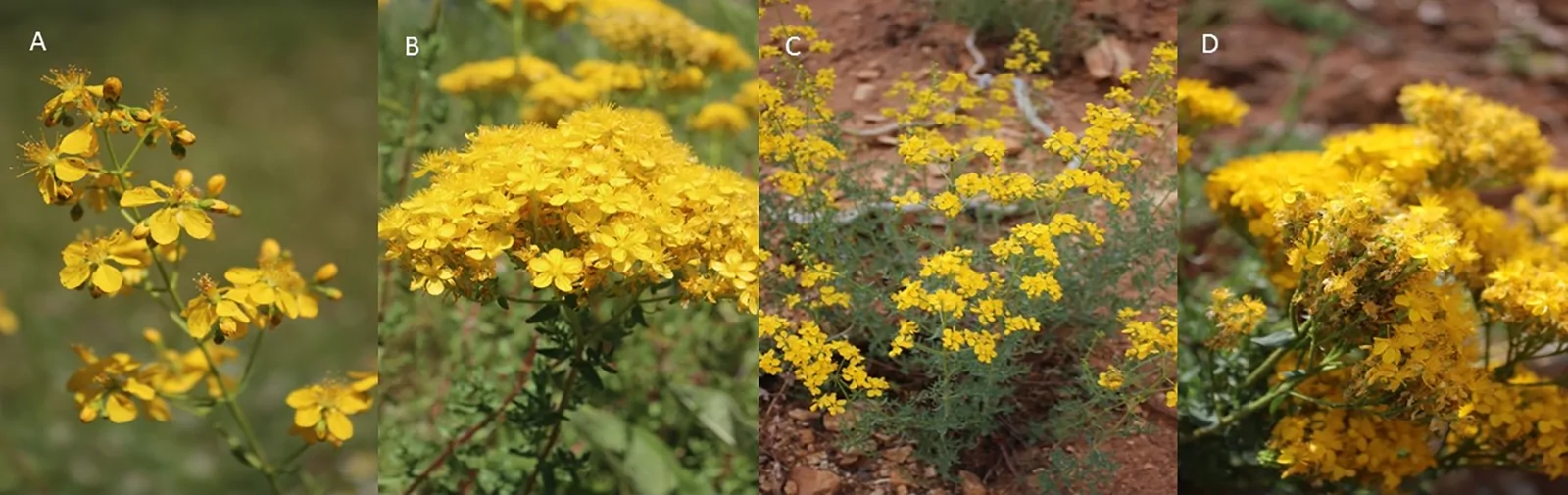
Photographs of *H. lydium* **(A)** and *H. scabrum* **(B)** collected from the non-mining area, and *H. lydium* **(C)** and *H. scabrum* **(D)** collected from the mining area, in their natural habitats.

The selection of Al, Cd, Cr, Fe, and Mn for heavy metal analysis was based on the geological characteristics of the study area, previous environmental investigations, and the objectives of the present study. The Avnik iron mine is located in the Bingöl–Genç district, which is characterized by important metallic mineral deposits. Previous geological investigations have reported that metallic mineral deposits, including iron (Fe), lead-zinc (Pb-Zn), phosphate, and kyanite occurrences, are mainly distributed in the Bingöl–Genç district and surrounding areas (Şahin and İnci, 2026a). In addition, previous studies conducted in Bingöl Province have reported the presence and distribution of several potentially toxic elements in soils, including Cr, Cd, Fe, and Mn (Taş and Demir, 2022). Furthermore, investigations on plant species growing around the Avnik iron mine have demonstrated the importance of Al, Cr, Fe, and Mn accumulation in native vegetation exposed to mining environments (Kırat and Vural, 2024). Therefore, these five elements were selected as representative indicators to evaluate metal accumulation, organ-specific distribution, and the potential effects of mining activities on the studied *Hypericum* species. Specimens of *H. scabrum* and *H. lydium* were collected from both sampling sites (**Figure 1**). Species identification was conducted in accordance with Flora of Turkey and the East Aegean Islands (Davis, 1966), and all specimens were documented photographically. During sampling, all relevant plant organs (including roots, stems, leaves, and flowers) were carefully collected to ensure accurate taxonomic identification as well as to enable subsequent elemental and fatty acid analyses. A total of 60 individual plant specimens were collected from the two sampling sites, including 30 individuals from the mining-impacted area and 30 individuals from the reference area. At each site, 15 individuals of *H. lydium* and 15 individuals of *H. scabrum* were collected. Following collection, each plant specimen was separated into four different organs: roots, stems, leaves, and flowers. The collected plant materials were washed, oven-dried, and ground into a fine powder prior to analysis. For each species and sampling site, the same plant organs obtained from the 15 individual specimens were pooled and homogenized to obtain representative composite samples. Accordingly, separate root, stem, leaf, and flower composite samples of *H. lydium* and *H. scabrum* from both the mining-impacted and reference sites were prepared and subjected to subsequent elemental and fatty acid analyses, while flower samples were also used for palynological evaluations. This sampling approach enabled the assessment of organ-specific differences while providing representative information for each species and sampling site.

### 2.2 Analysis of plant fatty acid composition

Extraction of Oils and Preparation of Fatty Acid Methyl Esters (FAMEs): Dried plant materials, including roots, stems, leaves, and flowers, were finely powdered using a ball mill. Total lipids were then isolated using a hexane–isopropanol mixture (3:2, v/v) following the procedure of Hara and Radin (1978). The lipid extracts were subjected to centrifugation at (correct value to be inserted) for 10 minutes and subsequently filtered. Solvent removal was performed under reduced pressure with a rotary evaporator maintained at 50 °C. Fatty acids were esterified to their methyl esters using 2% sulfuric acid in methanol (Christie, 1989), and the resulting FAMEs were extracted with 2.5 mL hexane prior to chromatographic evaluation.

Gas Chromatography Analysis: The fatty acid methyl esters were examined using a gas chromatograph coupled with flame ionization detection and mass spectrometry (Agilent 7890A GC with a 5975C MS detector). Separation was performed on a BPX90 capillary column, with nitrogen serving as the carrier gas at a flow rate of 3 mL min^−1^. The injector and detector temperatures were maintained at (120–250°C and 230–270°C, respectively). Identification of individual fatty acids was carried out by comparison with authentic standards and further verified using the NIST and Wiley mass spectral libraries. The oven temperature program was set as follows: an initial hold at 50°C for 2 min, ramping to 200°C at 20°C min^−1^, followed by an increase to 230°C at 5°C min^−1^, with a final hold for 30 min. The total runtime for the analysis was 55.5 minutes.

### 2.3 Heavy metal (Al, Cd, Cr, Fe, and Mn) analyses in plant and soil

Within the scope of heavy metal analyses, plant samples were collected during field studies together with all vegetative and generative organs, and subsequently separated into root, stem, leaf, and flower tissues under laboratory conditions. To minimize surface contamination, the samples were washed with ultrapure water and then oven-dried at 70 °C until constant weight was achieved. The dried plant material was homogenized by grinding with a hand mill to prepare it for analysis.

For the determination of total element content, microwave-assisted acid digestion was performed in accordance with the procedure described in the literature (Miller, 1998). Following digestion, the resulting solutions were filtered through filter paper, transferred into tubes, and their volumes were adjusted to 50 mL with ultrapure water. The concentrations of Al, Cd, Cr, Fe, and Mn in the diluted samples were determined using Inductively Coupled Plasma–Mass Spectrometry (ICP-MS). During the analytical process, instrument calibrations were performed in accordance with quality control procedures, and measurements were carried out within acceptable limits of analytical accuracy and precision.

### 2.4 Palynological analysis

Within the scope of palynological analyses, pollen grains obtained from mature anthers of *H. lydium* and *H. scabrum* individuals collected from the mining site and the Bingöl University campus (non-mining site) were examined. Pollen preparations for light microscopy (LM) were prepared following the method described by Woodhouse (1935). Morphological measurements and microphotographic documentation were performed using an OLYMPUS CX31 light microscope equipped with a digital camera.

**Figure 2:**
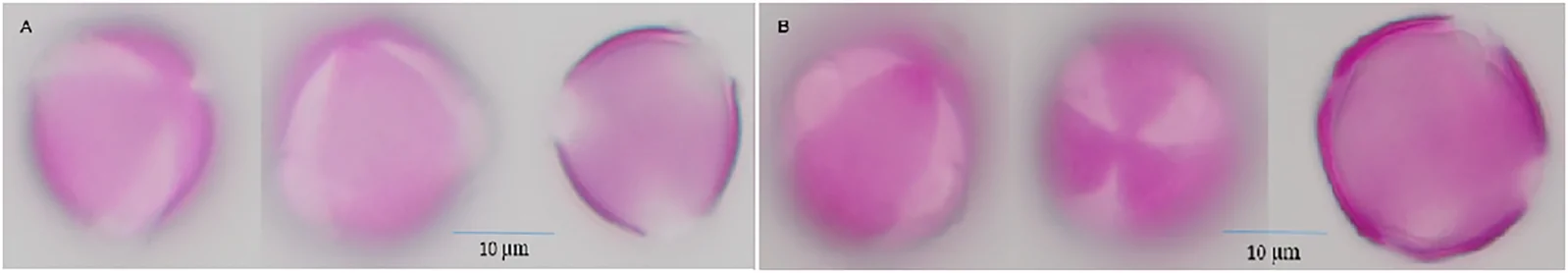
Light microscopy images of pollen grains of *Hypericum* species [**(A)** *H. lydium*; **(B)** *H. scabrum*].

During the analyses, key palynological characters [including polar axis length (P), equatorial axis length (E), P/E ratio, pollen shape, exine thickness (Ex), and intine thickness (In)] were evaluated. For each palynological parameter, at least 30 replicate measurements were conducted (**Figure 2**).

### 2.5 Statistical analysis

The relationship between heavy metal accumulation in plant organs and saturated and unsaturated fatty acid contents was evaluated using Pearson’s correlation analysis (**Table 2**). All statistical analyses were performed using IBM SPSS Statistics v.27 software. Morphological measurements of pollen were first subjected to the Shapiro–Wilk normality test. Subsequently, differences observed between populations from mining and non-mining areas of the same species were assessed for statistical significance using an independent samples t-test (**Table 3**). In all statistical evaluations, a significance level of p < 0.05 was adopted, corresponding to a 95% confidence interval.

**Table 2:** Correlation coefficients between heavy metal accumulation in plant organs and saturated and unsaturated fatty acid components.

		Al	Cd	Cr	Fe	Mn
*H. scabrum*	TSFA	0,76	0,28	0,86	0,70	0,76
	TUSFA	-0,76	-0,28	-0,86	-0,70	-0,76
		strong	weak	strong	moderate	strong
*H. lydium*	TSFA	0,58	0,10	0,66	0,58	0,58
	TUSFA	-0,58	-0,10	-0,66	-0,58	-0,58
		moderate	very strong	moderate	moderate	moderate

**Table 3:** Comparison of pollen characteristics between mining-site and control populations of *H. scabrum* and *H. lydium* using independent samples t-test.

	*H. scabrum*	*H. lydium*
	Sig. (2-tailed)	Sig. (2-tailed)
polar_axis	0,88	0,00
equatorial_axis	0,84	0,00
exine	0,00	0,01
intine	0,07	0,26

The experimental design was based on comparisons among two environmental conditions (mining-impacted and reference sites), two *Hypericum* species (*H. scabrum* and *H. lydium*), and different plant organs (root, stem, leaf, and flower). The effects of mining-related environmental conditions were evaluated by comparing heavy metal accumulation, fatty acid composition, and pollen morphological characteristics between populations. This comparative approach enabled the assessment of species-specific responses to heavy metal stress under natural field conditions.

### 2.6 Translocation factor (TF)

The translocation factor (TF) is commonly used to evaluate the ability of plants to transport accumulated metals from roots to aboveground tissues. A TF value greater than 1 indicates effective root-to-shoot translocation and suggests a potential capacity for metal accumulation, which is an important characteristic in phytoremediation studies (Sürmen et al., 2019). TF values were calculated according to the equation described by (Ortakcı, 2020) and (Gökdere et al., 2025).

## 3 Results

### 3.1 Comparative analysis of Al, Cd, Cr, Fe, and Mn distribution in plant organs of *H. scabrum* and *H. lydium* across two contrasting habitats

The variations in Al, Cd, Cr, Fe, and Mn concentrations determined in *H. scabrum* and *H. lydium* samples collected from two different sites, at both habitat and species levels, are presented in **Table 4** as follows:

**Table 4:** Distribution of Al, Cd, Cr, Fe and Mn concentrations in plant organs of *H. scabrum* and *H. lydium* collected from the mining site and the reference area.

Al (mg kg^-1^)	*Hypericum scabrum*		*Hypericum lydium*	
	Mining site	Non-mining site	Mining site	Non-mining site
Root	3034	523	517	406
Stem	929	43	131	63
Leaf	2580	108	438	110
Flower	3824	76	871	111
Mean	2592	187	489	172

Cd (mg kg^-1^)	*Hypericum scabrum*		*Hypericum lydium*	
	Mining site	Non-mining site	Mining site	Non-mining site
Root	0.41	0.38	0.39	0.38
Stem	0.52	0.47	0.67	0.47
Leaf	1.01	0.54	0.72	0.54
Flower	0.66	0.61	1.29	0.61
Mean	0.65	0.50	0.77	0.50

Cr (mg kg^-1^)	*Hypericum scabrum*		*Hypericum lydium*	
	Mining site	Non-mining site	Mining site	Non-mining site
Root	33.7	3.7	5.1	1.2
Stem	11.4	0.2	1.3	0.5
Leaf	19.4	0.3	2.5	0.4
Flower	13.3	0.4	4.2	0.5
Mean	19.5	1.2	3.3	0.7

Fe (mg kg^-1^)	*Hypericum scabrum*		*Hypericum lydium*	
	Mining site	Non-Mining Site	Mining Site	Non-Mining Site
Root	2680	608	517	406
Stem	995	47	131	63
Leaf	2858	124	438	110
Flower	4782	88	871	111
Mean	2829	217	489	172

Mn (mg kg^-1^)	*Hypericum scabrum*		*Hypericum lydium*	
	Mining site	Non-mining site	Mining site	Non-mining site
Root	62.0	26.0	17.5	8.8
Stem	28.6	10.1	14.1	6.6
Leaf	99.0	46.0	48.6	37.2
Flower	87.2	26.4	54.2	18.7
Mean	69.2	27.1	33.6	17.8

Al concentrations determined in *H. scabrum* and *H. lydium* samples collected from mining-impacted and reference sites exhibited pronounced differences both between species and across habitats.

In *H. scabrum*, the mean Al concentration was 2592 mg kg^−1^ in the mining site, compared to 187mg kg^−1^ in the reference site, indicating an approximately 14-fold increase under mining conditions. In *H. scabrum*, Al concentration in root tissues was 3034 mg kg^−1^ in the mining site and 523mg kg^−1^ in the reference site, representing an approximately 6-fold increase. Stem tissues exhibited Al concentrations of 929 mg kg^−1^ in the mining site and 43 mg kg^−1^ in the reference site, corresponding to an approximate 22-fold increase. In leaf tissues, Al levels were 2580 mg kg^−1^ in the mining area and 108 mg kg^−1^ in the reference area, representing an ≈24-fold increase. In flower tissues, Al concentrations reached 3824 mg kg^−1^ in the mining site and 76 mg kg^−1^ in the reference site, representing an approximately 50-fold increase (**Table 4**).

In *H. lydium*, the mean Al concentration was 489 mg kg^−1^ in the mining site and 172 mg kg^−1^ in the reference site. This corresponds to an approximately 3fold increase, which is considerably lower compared to *H. scabrum.* In *H. lydium*, Al concentrations in root tissues were 517 mg kg^−1^ in the mining site and 406 mg kg^−1^ in the reference site, corresponding to an approximately 1-fold increase. In stems, concentrations of 131 mg kg^−1^ (mining) and 63mg kg^−1^ (reference) were recorded, representing a 2-fold increase. Leaf tissues exhibited 438 mg kg^−1^ in the mining site and 110mg kg^−1^ in the reference site, indicating an approximate 4-fold increase. In flowers, Al levels reached 871 mg kg^−1^ (mining) and 111 mg kg^−1^ (reference), corresponding to an 8-fold increase (**Table 4**).

Cd concentrations determined in *H. scabrum* and *H. lydium* samples collected from the mining and reference sites varied depending on both habitat and species. Overall, Cd levels in both species tended to increase in the mining site compared to the reference site; however, this increase was considerably more limited compared to Al accumulation.

In *H. scabrum*, the mean Cd concentration was 0.65 mg kg^−1^ in the mining site and 0.50 mg kg^−1^ in the reference site, representing an approximately 1-fold increase. Organ-specific analysis showed Cd levels of 0.41 mg kg^−1^ (mining) and 0.38 mg kg^−1^ (reference) in roots (1-fold increase), and 0.52 mg kg^−1^ (mining) versus 0.47 mg kg^−1^ (reference) in stems (1-fold increase). In leaves, concentrations were 1.01 mg kg^−1^ in the mining site and 0.54 mg kg^−1^ in the reference site, corresponding to an 1.9-fold increase. Flower tissues exhibited 0.66 mg kg^−1^ (mining) and 0.61 mg kg^−1^ (reference), reflecting a 1-fold increase. These results indicate that Cd accumulation in *H. scabrum* is particularly pronounced in leaf tissues (**Table 4**).

In *H. lydium*, the mean Cd concentration was 0.77 mg kg^−1^ in the mining site and 0.50 mg kg^−1^ in the reference site, corresponding to an approximately 2-fold increase. Organ-specific measurements indicated Cd levels of 0.39 mg kg^−1^ (mining) and 0.38 mg kg^−1^ (reference) in roots (1-fold increase), and 0.67 mg kg^−1^ (mining) versus 0.47 mg kg^−1^ (reference) in stems (1-fold increase). In leaves, concentrations were 0.72 mg kg^−1^ (mining) and 0.54 mg kg^−1^ (reference), representing a 1-fold increase. Flower tissues exhibited 1.29 mg kg^−1^ in the mining site and 0.61 mg kg^−1^ in the reference site, reflecting an 2-fold increase. The pronounced accumulation in flowers indicates that Cd is translocated to reproductive organs to a greater extent in *H. lydium* (**Table 4**).

Cr concentrations determined in *H. scabrum* and *H. lydium* samples collected from the mining and reference sites exhibited pronounced differences depending on both habitat and species. In both species, Cr accumulation in individuals from the mining site was markedly higher compared to those from the reference site.

In *H. scabrum*, the mean Cr concentration was 19.5 mg kg^−1^ in the mining site and 1.2 mg kg^−1^ in the reference site, corresponding to an approximately 16-fold increase. Organ-specific analysis showed Cr levels of 33.7 mg kg^−1^ (mining) and 3.7 mg kg^−1^ (reference) in roots (9-fold increase), and 11.4 mg kg^−1^ (mining) versus 0.4 mg kg^−1^ (reference) in stems (29-fold increase). Leaf tissues exhibited 19.4 mg kg^−1^ (mining) and 0.3 mg kg^−1^ (reference), representing an ≈65-fold increase. In flowers, Cr concentrations were 13.3 mg kg^−1^ in the mining site and 0.4 mg kg^−1^ in the reference site, corresponding to an 33-fold increase. These results indicate that Cr accumulation in *H. scabrum* is particularly pronounced in aboveground organs (**Table 4**).

In *H. lydium*, the mean Cr concentration was 3.1 mg kg^−1^ in the mining site and 0.7 mg kg^−1^ in the reference site, corresponding to an approximately 5-fold increase. Organ-specific measurements indicated Cr levels of 5.1 mg kg^−1^ in roots (mining) and 1.2 mg kg^−1^ (reference), reflecting a 4-fold increase. In stems, Cr concentrations were 1.3 mg kg^−1^ (mining) and 0.5 mg kg^−1^ (reference), representing a 2.51-fold increase. Leaves exhibited 2.5 mg kg^−1^ (mining) and 0.4 mg kg^−1^ (reference), corresponding to a 6-fold increase. Flower tissues showed 4.2 mg kg^−1^ (mining) and 0.5 mg kg^−1^ (reference), indicating a 9-fold increase (**Table 4**).

Fe concentrations determined in *H. scabrum* and *H. lydium* samples from the mining and reference sites exhibited pronounced differences depending on both habitat and species. In both species, Fe accumulation in individuals from the mining site was substantially higher compared to those from the reference site.

In *H. scabrum*, the mean Fe concentration was 2829 mg kg^−1^ in the mining site and 217 mg kg^−1^ in the reference site, representing an approximate 13-fold increase. Organ-specific analysis revealed Fe levels of 2680 mg kg^−1^ in roots (mining) and 608 mg kg^−1^ (reference), corresponding to a 4-fold increase. In stems, concentrations were 995 mg kg^−1^ (mining) and 47 mg kg^−1^ (reference), indicating a 21-fold increase. Leaves exhibited 2858 mg kg^−1^ (mining) and 124 mg kg^−1^ (reference), reflecting an 23-fold increase. Flower tissues showed 4782 mg kg^−1^ (mining) and 88 mg kg^−1^ (reference), corresponding to an ≈54-fold increase. Notably, the substantial accumulation observed in leaf and flower tissues is particularly remarkable (**Table 4**).

In *H. lydium*, the mean Fe concentration was 489 mg kg^−1^ in the mining site and 172 mg kg^−1^ in the reference site, indicating an approximate 3-fold increase. Organ-specific measurements showed Fe levels of 517 mg kg^−1^ in roots (mining) and 406 mg kg^−1^ (reference), corresponding to a 1-fold increase. In stems, concentrations were 131 mg kg^−1^ (mining) and 63 mg kg^−1^ (reference), representing a 2-fold increase. Leaves exhibited 438 mg kg^−1^ (mining) and 110 mg kg^−1^ (reference), reflecting a 4-fold increase. Flower tissues contained 871mg kg^−1^ (mining) and 111 mg kg^−1^ (reference), corresponding to an approximately 8-fold increase (**Table 4**).

Mn concentrations in *H. scabrum* and *H. lydium* samples collected from the mining and reference sites exhibited clear differences at both habitat and species levels. In both species, individuals collected from the mining site generally accumulated higher Mn concentrations than those from the reference site. In *H. scabrum*, the mean Mn concentration was 69.2 mg kg^−1^ in the mining site and 27.1 mg kg^−1^ in the reference site, corresponding to an approximately 3-fold increase under mining conditions. Organ-specific analysis revealed Mn concentrations of 62 mg kg^−1^ in roots from the mining site and 26 mg kg^−1^ in those from the reference site, representing a 2-fold increase. Stem tissues contained 28.6 mg kg^−1^ (mining) and 10.1 mg kg^−1^ (reference), corresponding to a 3-fold increase. Leaf tissues exhibited Mn concentrations of 99 mg kg^−1^ in the mining site and 46 mg kg^−1^ in the reference site, representing a 2-fold increase. Flower tissues contained 87.2 mg kg^−1^ (mining) and 26.2 mg kg^−1^ (reference), corresponding to an approximately 3-fold increase. Among the analyzed organs, the highest Mn concentration was detected in leaves, followed by flowers (**Table 4**).

In *H. lydium*, the mean Mn concentration was 33.6 mg kg^−1^ in the mining site and 17.8 mg kg^−1^ in the reference site, corresponding to an approximately 2-fold increase. Organ-specific analysis showed Mn concentrations of 17.5 mg kg^−1^ in roots from the mining site and 8.8 mg kg^−1^ in those from the reference site, indicating a 2-fold increase. Stem tissues contained 14.1 mg kg^−1^ (mining) and 6.6 mg kg^−1^ (reference), representing a 2-fold increase. Leaf tissues exhibited 48.6 mg kg^−1^ (mining) and 37.2 mg kg^−1^ (reference), corresponding to a 1-fold increase. Flower tissues contained 54.2 mg kg^−1^ in the mining site and 18.7 mg kg^−1^ in the reference site, representing an approximately 3-fold increase. The highest Mn concentrations were observed in flower and leaf tissues, suggesting relatively greater Mn accumulation in the aerial organs of *H. lydium* (**Table 4**).

### 3.2 Translocation factor (TF) for the species across two different habitats

In this study, the Translocation Factor (TF) values for Al, Cd, Cr, Fe, and Mn were calculated for *H. scabrum* and *H. lydium* collected from both the mining site and the non-mining area. The results revealed pronounced differences in metal translocation strategies between the species and across the sites. TF values greater than 1 indicate efficient translocation of the respective metal from root to shoot, whereas values below 1 suggest that the metal is predominantly retained in the roots. These findings demonstrate that both species have developed distinct physiological response mechanisms to cope with metal stress. The TF values for Al, Cd, Cr, Fe, and Mn in *H. scabrum* and *H. lydium* collected from the mining and non-mining areas are presented in **Table 5**.

**Table 5:** Translocation Factor (TF) values for *H. scabrum* and *H. lydium* collected from the mining and non-mining areas.

Species	Area	TF_Al_	TF_Cd_	TF_Cr_	TF_Fe_	TF_Mn_
*H. scabrum*	Mining Site	0.81	1.79	0.44	1.07	1.15
	Non-Mining Site	0.14	1.44	0.10	0.14	1.06
*H. lydium*	Mining Site	0.93	2.30	0.53	1.93	2.23
	Non-Mining Site	0.23	1.08	0.39	0.54	2.37

For *H. scabrum* collected from the mining site, the TF values were determined as follows: Al (0.81), Cd (1.79), Cr (0.44), Fe (1.07), and Mn (1.15). In contrast, the TF values for the same species from the non-mining area were Al (0.14), Cd (1.44), Cr (0.10), Fe (0.14), and Mn (1.06). TF values at the mining site were approximately 6-, 8-, and 4-fold higher for Al, Fe, and Cr, respectively, compared to the non-mining area. For Cd, the TF value at the mining site showed an increase of approximately 1-fold, while Mn values were similar across both sites, with a slight increase observed in the mining area (**Table 5**).

For *H. lydium*, the TF values at the mining site were calculated as Al (0.93), Cd (2.30), Cr (0.53), Fe (1.93), and Mn (2.23), whereas at the non-mining site, the corresponding values were Al (0.23), Cd (1.08), Cr (0.39), Fe (0.54), and Mn (2.37). At the mining site, TF values were approximately 4-, 2-, and 4-fold higher for Al, Cd, and Fe, respectively, compared to the non-mining area. The TF value for Cr was approximately 1-fold higher at the mining site. For Mn, the TF value at the non-mining site (2.37) was slightly higher than that at the mining site (2.23) (**Table 5**).

In the interspecific comparison at the mining site, *H. lydium* exhibited higher TF values *than H. scabrum* for all examined metals. For Al, TF in *H. lydium* was 0.93, compared to 0.81 *in H. scabrum*. For Cd, the values were 2.30 and 1.79; for Cr, 0.53 and 0.44; for Fe, 1.93 and 1.07; and for Mn, 2.23 and 1.15, respectively. In all cases, TF values in *H. lydium* were higher than those in *H. scabrum* (**Table 5**).

Similarly, in the non-mining area, *H. lydium* exhibited higher TF values for most metals compared to *H. scabrum*. The TF value for Al was 0.23, approximately 2-fold higher than that of *H. scabrum* (0.14). For Cd, the TF value in *H. lydium* was 1.08, lower than *H. scabrum* (1.44; ~1-fold). TF values for Cr (0.39 vs. 0.10) and Fe (0.54 vs. 0.14) were approximately 4-fold higher in *H. lydium*. Mn values were relatively similar between the two species, with H. *lydium* (2.37) approximately 2-fold higher than *H. scabrum* (1.06) (**Table 5**).

### 3.3 Comparative analysis of fatty acid composition in *Hypericum* species across different sites

**Figure 3:**
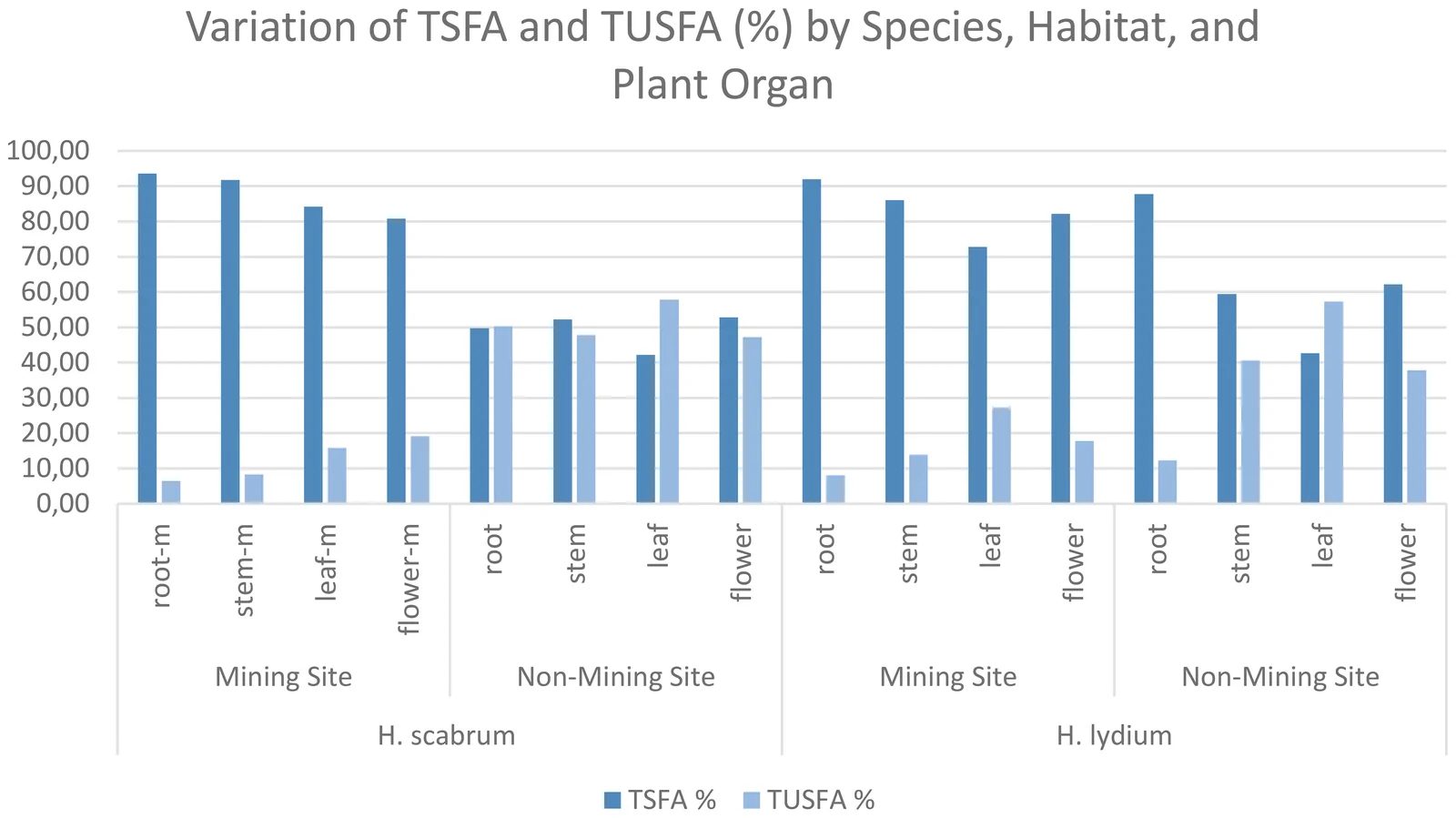
Habitat-Dependent Variation in Saturated (TSFA) and Unsaturated (TUSFA) Fatty Acid Ratios in *H. scabrum* and *H. lydium*.

The total saturated fatty acid (ΣTSFA) and total unsaturated fatty acid (ΣTUSFA) contents in root, stem, leaf, and flower tissues of *Hypericum scabrum* and *Hypericum lydium* (**Table 6**; **Figure 3**) under mining area and non-mining area conditions are as follows:

**Table 6:** Fatty acid composition (%) of *H. scabrum* and *H. lydium* collected from mining and non-mining sites.

	Mining site				Non-mining site				Mining site				Non-mining site			
	*Hypericum scabrum*								*Hypericum lydium*							
	Root	Stem	Leaf	Flower	Root	Stem	Leaf	Flower	Root	Stem	Leaf	Flower	Root	Stem	Leaf	Flower
C 8:0								2.24				1.02				1.99
C 10:0							1.44	1.57				1.41				1.23
C 12:0			3.49	1.79		0.15	0.73	2.96			1.49	3.40			0.81	2.90
C 13:0							1.79									
C 14:0	1.92	1.76	3.55	5.22	1.39		1.22				3.02	2.16			1.23	1.36
C 15:0	1.18			0.60	0.98	0.75	0.30	0.44	2.04	1.12	0.50	0.64	2.27	1.30	0.28	0.41
C 16:0	50.88	50.77	43.87	40.21	26.53	32.69	19.17	26.08	54.73	51.05	39.89	40.52	55.32	40.32	19.72	27.22
C 17:0	1.37	1.06		1.22	0.83	0.84	0.17	0.19	2.47	1.53	0.91	1.52	1.79	1.11	0.40	1.34
C 18:0	31.01	24.42	19.10	24.31	12.99	10.21	7.53	11.48	22.60	23.04	15.73	21.48	22.82	12.98	7.71	13.71
C 20:0	2.00	1.30	2.29	2.00	1.48	0.97	1.40	1.86	1.93	2.12	3.09	3.33	1.62	1.39	1.78	2.27
C 21:0						0.88			0.79	0.84	1.25	1.13		0.78		1.19
C 22:0	1.87	5.59	4.45	3.42	2.40	2.84	3.46	4.85	3.56	3.70	4.08		2.50		7.04	5.29
C 23:0	1.45	2.93	3.57		2.03	2.90		1.14	2.34	1.86	1.21	1.79	1.40	1.58	1.23	1.21
C 24:0	1.92	3.92	3.86	2.07	1.09		4.99		1.52	0.82	1.62	3.79			2.49	2.06
∑TSFA	93.60%	91.75%	84.18%	80.84%	49.72%	52.23	42.19%	52.81%	91.98%	86.08%	72.79%	82.19%	87.72%	59.46%	42.69%	62.18%
C 16:1			2.11		0.41										1.46	1.05
C 18:1	3.69	1.56	3.19	9.26	14.84	4.26	4.51	6.44	3.20	3.26	4.71	5.63	5.62	12.25	6.95	3.87
C 18:2	2.71	4.75	3.35	4.78	18.66	21.49	6.98	11.21	3.67	5.89	3.35	4.17	3.90	16.34	5.49	4.90
C 18:3		1.94	7.17	5.12	6.43	21.11	45.69	27.08	1.15	3.67	19.15	8.01	2.76	11.95	40.20	26.12
C 20:1					1.53	0.91		0.72		1.10						
C 20:2					0.57		0.17	0.25								
C 20:3							0.46	1.49							3.21	1.88
C 22:1					7.84											
∑TUSFA	6.40%	8.25%	15.82%	19.16%	50.28%	47.77%	57.81%	47.19%	8.02%	13.92%	27.21%	17.81%	12.28%	40.54%	57.31%	37.82%
																

(C8:0, Caprylic acid; C10:0, Capric acid; C12:0, Lauric acid; C13:0, Tridecanoic acid; C14:0, Myristic acid; C15:0, Pentadecanoic acid; C16:0, Palmitic acid; C17:0, Heptadecanoic acid; C18:0, Stearic acid; C20:0, Arachidic acid; C21:0, Heneicosanoic acid; C22:0, Behenic acid; C23:0, Tricosanoic acid; C24:0, Lignoceric acid C16:1, Palmitoleic acid;C18:1, Oleic acid; C18:2, Linoleic acid; C18:3, Linolenic acid; C20:1, Eicosanoic acid; C20:2, Eicosadienoic acid; C20:3, Eicosatrienoic acid; C22:1, Docosaenoic acid; SFA, Saturated fatty acid; USFA, Unsaturated fatty acid).

In *H. scabrum*, the ΣTSFA content in the mining area was 93.60% in roots, 91.75% in stems, 84.18% in leaves, and 80.84% in flowers. Corresponding ΣTUSFA values were 6.40%, 8.25%, 15.82%, and 19.16%, respectively. In contrast, in the non-mining area, ΣTSFA levels were 49.72% in roots, 52.23% in stems, 42.19% in leaves, and 52.81% in flowers, while ΣTUSFA contents were 50.28%, 47.77%, 57.81%, and 47.19%, respectively. Notably, the proportion of saturated fatty acids was significantly higher in roots under mining site conditions, whereas unsaturated fatty acids predominated in roots collected from the non-mining area (**Table 6**).

In *H. lydium*, the ΣTSFA content in the mining area was 91.98% in roots, 86.08% in stems, 72.79% in leaves, and 82.19% in flowers, while the corresponding ΣTUSFA values were 8.02%, 13.92%, 27.21%, and 17.81%, respectively. In the non-mining area, ΣTSFA levels were 87.72% in roots, 59.46% in stems, 42.69% in leaves, and 62.18% in flowers, whereas ΣTUSFA contents were 12.28%, 40.54%, 57.31%, and 37.82%, respectively. Notably, unsaturated fatty acids were present at higher proportions in leaves collected from the non-mining area (**Table 6**).

In both species, palmitic acid (C16:0) and stearic acid (C18:0) were identified as the predominant saturated fatty acids. The changes in these acids under mining site conditions varied depending on both the species and the specific organ analyzed.

Regarding palmitic acid (C16:0), *H. scabrum* exhibited notable increases under mining site conditions. In roots, the content rose to 50.88% compared to 26.53% in the non-mining area, representing an approximate 2-fold increase. Similarly, stems increased to 50.77% versus 32.69% (2-fold), leaves to 43.87% versus 19.17% (2-fold), and flowers to 40.21% versus 26.08% (2-fold).

In *H. lydium*, changes in roots were minimal (54.73% vs. 55.32%, ~1-fold), and stems (51.05% vs. 40.32%) showed no substantial differences. However, in leaves, palmitic acid content was higher in the mining area (39.89%) compared to the non-mining area (19.72%, 2-fold), and in flowers, it increased from 27.22% to 40.52% (1-fold) (**Table 6**).

Regarding stearic acid (C18:0), both species generally exhibited higher proportions under mining site conditions. In *H. scabrum*, stearic acid content in roots, stems, leaves, and flowers was 31.01%, 24.42%, 19.10%, and 24.31%, respectively, which are markedly higher than the corresponding values in the non-mining area (roots 12.99%, stems 10.21%, leaves 7.53%, and flowers 11.48%). Similarly, in *H. lydium*, higher stearic acid levels were observed in roots (22.60%), stems (23.04%), leaves (15.73%), and flowers (21.48%) at the mining site. In the non-mining area, decreases were particularly evident in stems (12.98%), leaves (7.71%), and flowers (13.71%), whereas root levels remained similar (22.82%) (**Table 6**).

In *H. scabrum*, an increasing trend was observed across all tissues under mining site conditions, with approximately 2-fold increases in roots, stems, leaves, and flowers. In *H. lydium*, changes in roots were negligible (~1-fold), whereas increases of 1-fold in stems, 2-fold in leaves, and 2-fold in flowers were observed. Overall, palmitic and stearic acid levels were higher under mining site conditions in both species, with the increases being more pronounced in *H. scabrum*.

In contrast, the major unsaturated fatty acids (oleic acid (C18:1), linoleic acid (C18:2), and α-linolenic acid (C18:3)) were generally present at higher levels in samples from the non-mining area in both species.

Regarding oleic acid (C18:1), in *H. scabrum*, the content in the mining area was 3.69% in roots, 1.56% in stems, 3.19% in leaves, and 9.26% in flowers. In the non-mining area, values were notably higher, with 14.84% in roots, 4.26% in stems, 4.51% in leaves, and 6.44% in flowers. Fold increases were calculated as 3 in stems, 1 in leaves, and flowers. In *H. lydium*, oleic acid levels in the mining area were 3.20% in roots, 3.26% in stems, 4.71% in leaves, and 5.63% in flowers, whereas in the non-mining area, the contents were 5.62%, 12.25%, 6.95%, and 3.87%, respectively. The fold increases in the non-mining samples were 2 in roots, 4 in stems, 1 in leaves, and 2 in flowers (**Table 6**).

Regarding linoleic acid (C18:2), pronounced differences were observed in roots and stems of both species. In *H. scabrum*, the content in the mining area was 2.71% in roots, 4.75% in stems, 3.35% in leaves, and 4.78% in flowers. In the non-mining area, these values increased markedly to 18.66%, 21.49%, 6.98%, and 11.21%, respectively. Fold increases in the non-mining samples were 7 in roots, 5 in stems, 2 in leaves, and 2 in flowers. Similarly, in *H. lydium*, linoleic acid levels in the mining area were 3.67% in roots, 5.89% in stems, 3.35% in leaves, and 4.17% in flowers. In the non-mining area, values were 3.90%, 16.34%, 5.49%, and 4.90%, respectively, with a particularly notable increase in stems under non-mining conditions (**Table 6**).

Regarding α-linolenic acid (C18:3), pronounced differences were observed, particularly in leaf tissues. In *H. scabrum*, the content in the mining area was 1.94% in stems, 7.17% in leaves, and 5.12% in flowers, whereas in the non-mining area, considerably higher levels were detected: 6.43% in roots, 21.11% in stems, 45.69% in leaves, and 27.08% in flowers. Leaf samples from the non-mining area contained 45.69% α-linolenic acid, representing a 6-fold increase compared to the mining site, and flowers showed a 5-fold increase. In *H. lydium*, α-linolenic acid levels in the mining area were 1.15% in roots, 3.67% in stems, 19.15% in leaves, and 8.01% in flowers. In the non-mining area, the contents increased to 2.76%, 11.95%, 40.20%, and 26.12%, respectively, with fold increases of 2 in leaves and 3 in flowers (**Table 6**).

Overall, unsaturated fatty acids were present at higher levels under non-mining conditions in both species, with this increase being particularly pronounced in *H. scabrum*.

### 3.4 Comparative palynological analysis of *Hypericum* species from contrasting sites

A comparative palynological analysis was conducted for *Hypericum scabrum* and *Hypericum lydium* using specimens collected from mining and non-mining sites (**Table 7**).

**Table 7:** Comparison of pollen characteristics (µm) of *H. scabrum* and *H. lydium* collected from mining and non-mining sites (SD, Standard deviation; P, Polar axis; E, Equatorial axis).

*H. scabrum*	Non- mining site					
	P	E	P/E	Pollen shape and size class	Exine	Intine
Mean	21.21	21.50	0.98	Oblatae-sphaeroidae	0.55	0.62
SD	0.65	0.86		Small -sized pollen grains	0.07	0.09

	Mining site					
	P	E	P/E	Pollen shape and size class	Exine	Intine
Mean	21.24	21.48	0.99	Oblatae-sphaeroidae	0.61	0.58
SD	1.15	1.06		Small -sized pollen grains	0.07	0.06
						

*H. lydium*	Non- mining site					
	P	E	P/E	Pollen shape and size class	Exine	Intine
Mean	15.32	14.67	1.04	Prolatae-sphaeroidae	0.46	0.43
SD	0.51	0.47		Small -sized pollen grains	0.06	0.06

	Mining site					
	P	E	P/E	Pollen shape and size class	Exine	Intine
Mean	16.23	14.13	1.15	Subprolatae	0.50	0.45
SD	0.60	0.56		Small -sized pollen grains	0.06	0.05

For *H. scabrum*, a slight increase was observed in the polar axis (1-fold) and P/E ratio (1.01-fold), whereas a limited decrease was detected in the equatorial axis (1-fold) and intine thickness (1-fold). In contrast, exine thickness exhibited an increase in samples from the mining site (1-fold). Despite these variations, pollen shape (oblate-spheroidal) and size class (small) remained unchanged across both environments. Overall, in *H. scabrum*, pollen size and morphology were largely conserved under mining site conditions, with only minor modifications observed in the wall layers (**Table 7**).

For *H. lydium*, comparison of specimens from mining and non-mining sites revealed an increase in the polar axis (1-fold) and P/E ratio (1-fold), accompanied by a decrease in the equatorial axis (1-fold). Correspondingly, pollen shape shifted from prolate-spheroidal to subprolate. While both exine (1-fold) and intine (1-fold) thicknesses increased, the size class remained consistently small across both environments. Overall, in *H. lydium*, mining site conditions were associated with increases in both the P/E ratio and wall thicknesses, alongside a shift toward a more prolate pollen morphology (**Table 7**).

### 3.5 Heavy metal accumulation, fatty acid composition, and pollen morphology

To provide a statistical evaluation of the relationships among the investigated parameters, correlation analysis and independent samples t-tests were performed. Correlation analysis was used to assess the associations between heavy metal accumulation and fatty acid composition at the species level, whereas independent samples t-tests were applied to determine whether differences in pollen morphological characteristics between mining-site and non-mining-site populations were statistically significant. The statistical results obtained from these analyses are presented in the following subsections.

#### 3.5.1 Correlation analysis

When the correlations between heavy metal concentrations accumulated in plant organs and fatty acid components were evaluated at the species level, in *H. scabrum*, strong positive correlations were observed between saturated fatty acids (TSFA) and Al (r = 0.76), Cr (r = 0.86), and Mn (r = 0.76). A moderate positive correlation was found with Fe (r = 0.70), whereas a weak positive relationship was detected with Cd (r = 0.28). In contrast, unsaturated fatty acids (TUSFA) showed inverse relationships of similar magnitude with the same metals (Al: r = −0.76; Cr: r = −0.86; Mn: r = −0.76; Fe: r = −0.70; Cd: r = −0.28). These findings indicate that in *H. scabrum*, increasing heavy metal accumulation is associated with an increase in the proportion of saturated fatty acids, while the proportion of unsaturated fatty acids decreases accordingly (**Table 2**).

For *H. lydium*, moderate positive correlations were identified between saturated fatty acids (TSFA) and Al (r = 0.58), Fe (r = 0.58), and Mn (r = 0.58). A moderate correlation was also observed with Cr (r = 0.66), whereas a very weak relationship was detected with Cd (r = 0.10). Unsaturated fatty acid (TUSFA) values exhibited negative correlations with all metals, with coefficients of similar magnitude to those of TSFA (Al r = −0.58; Cr r = −0.66; Fe r = −0.58; Mn r = −0.58; Cd r = −0.10). Overall, these results indicate that in *H. lydium*, heavy metal accumulation is positively associated with saturated fatty acids and negatively associated with unsaturated fatty acids. However, these relationships were generally weaker compared to those observed in *H. scabrum* (**Table 2**).

Based on the species-level correlation analysis, in *H. scabrum*, strong positive correlations were observed between saturated fatty acids (TSFA) and the elements Al, Cr, and Mn, while a moderate correlation was detected with Fe and a weak correlation with Cd. In the same species, an inverse relationship was identified between unsaturated fatty acids (TUSFA) and these elements, indicating that TUSFA content decreases as elemental concentrations increase. Furthermore, the strength of this relationship was found to be comparable to that observed for TSFA.

In *H. lydium*, TSFA showed generally moderate correlations with Al, Cr, Fe, and Mn, whereas a low-level relationship was observed with Cd. Similarly, an inverse relationship was found between TUSFA and all analyzed elements in this species, and the strength of this association was also comparable to that observed for TSFA.

#### 3.5.2 t-test for pollen morphology

The distribution of measurement data related to pollen characteristics was assessed using the Shapiro–Wilk normality test, and all variables were found to be normally distributed. Based on the independent samples t-test results presented in **Table 3**, differences in pollen morphological characteristics between mining-site and non-mining-site populations were evaluated at the species level in order to determine whether they were statistically significant. Accordingly;

For *H. scabrum*, the results indicate that there were no statistically significant differences between the groups in terms of polar axis (p = 0.88) and equatorial axis (p = 0.84) measurements (p > 0.05). Similarly, no significant difference was detected in intine thickness (p ≈ 0.07; p > 0.05). In contrast, a statistically significant difference was found in exine thickness between mining-site and non-mining-site samples (p = 0.00; p < 0.05). These findings suggest that in *H. scabrum*, heavy metal exposure particularly induces pronounced morphological changes in the exine layer (**Table 3**).

For *H. lydium*, both polar axis (p = 0.00) and equatorial axis (p = 0.00) values indicated highly significant differences between the groups (p < 0.001). Exine thickness also showed a statistically significant difference (p = 0.01; p < 0.05). In contrast, no significant difference was observed in intine thickness (p = 0.26; p > 0.05). These results demonstrate that the pollen morphology of *H. lydium* is more extensively affected by mining-site conditions, with pronounced changes occurring particularly in dimensional traits and exine structure. Overall, the findings indicate that the effect of mining-site conditions is relatively limited and specific in *H. scabrum*, being mainly restricted to exine-related alterations, whereas in *H. lydium* it is more widespread, affecting multiple pollen morphological traits (**Table 3**).

## 4 Discussion

### 4.1 Comparative analysis of Al, Cd, Cr, Fe, and Mn distribution in plant organs of *H. scabrum* and *H. lydium* across two contrasting habitats

In this study, the organ-specific distribution of Al, Cd, Cr, Fe, and Mn in *H. scabrum* and *H. lydium* samples collected from the mining and reference sites was comparatively evaluated. Overall, concentrations of all elements were generally higher in individuals from the mining site. However, the magnitude of increase varied markedly between species and plant organs. In *H. scabrum*, the accumulation of Al, Cr, and Fe was particularly pronounced, with increases reaching approximately 50-fold for Al in flowers, 65-fold for Cr in leaves, and 54-fold for Fe in flowers compared with the reference site. The most pronounced enrichment was generally observed in aerial and reproductive organs, particularly leaves and flowers, indicating substantial accumulation and translocation of these elements under mining conditions. In contrast, Mn showed a more moderate increase in *H. scabrum*, generally ranging from approximately 2- to 3-fold among the different organs. *H. lydium* exhibited a considerably lower degree of enrichment, although increases in Cr and Cd were also evident, particularly in flowers, with Cr showing the highest degree of enrichment among the analyzed organs. Overall, these findings demonstrate clear species- and organ-specific patterns of metal accumulation, with *H. scabrum* showing substantially greater enrichment of Al, Cr, and Fe, particularly in aerial and reproductive tissues, whereas *H. lydium* exhibited comparatively lower levels of metal accumulation. According to the literature, *Hypericum* species are considered among the plants capable of tolerating metal accumulation and, in some cases, are evaluated as potential phytoremediation species (Radanovic et al., 2022). Similarly, numerous studies have reported significant increases in metal content in medicinal plants growing in polluted areas. For instance, Kočevar Glavač et al. (2017) reported that the concentrations of heavy metals in tissues of medicinal plants cultivated in metal-contaminated regions were markedly higher compared to those from unpolluted areas, directly correlating with soil contamination.

Similar increases in heavy metal accumulation in plants exposed to mining-related environmental conditions have been reported in recent studies. Mining activities are recognized as important sources of soil metal contamination, increasing the availability of potentially toxic elements and consequently affecting metal uptake and distribution in plants (Sharma et al., 2023). Recent studies have emphasized that heavy metal accumulation patterns among plant organs are controlled not only by soil metal availability but also by species-specific physiological mechanisms, including root retention, uptake capacity, and internal translocation processes (Ghuge et al., 2023). Therefore, the differences observed between *H. scabrum* and *H. lydium* in the present study may be related to their distinct physiological strategies for metal uptake, sequestration, and tolerance.

When comparing the two species, *H. scabrum* exhibited a stronger accumulation response under mining conditions, whereas *H. lydium* showed a more limited but comparatively balanced increase. Although increases in all elements were observed in *H. lydium*, the magnitude of accumulation was lower than in *H. scabrum*. Metal accumulation can vary significantly among plant species. Indeed, studies on different *Hypericum* populations have also demonstrated that metal accumulation is dependent on both species and locality (Çolak et al., 2020).

When examined by organ, it was observed that metal accumulation in both species was not confined to the root tissues but increased markedly in leaf and flower tissues. This finding indicates that heavy metals can be translocated via the xylem to aerial organs and, in certain species, can accumulate substantially even in generative tissues. However, some studies have reported that metal accumulation predominantly occurs in vegetative tissues, with flowers generally exhibiting lower metal concentrations (Berber et al., 2021). In the present study, the elevated metal levels observed in the flower tissues of *H. scabrum* highlight the substantial metal load in the mining site and demonstrate that metal translocation extended to the generative organs.

When comparing the elements, it was evident that the increase in Cd was considerably more limited than that of the other metals. This can be attributed to the generally more controlled uptake of Cd by plants and its tendency to be retained predominantly in root tissues. The literature indicates that Cd is highly toxic to plants, and many species possess various physiological mechanisms that restrict its translocation to aerial organs (Hall, 2002; Clemens, 2006). Through these mechanisms, Cd is mostly sequestered in the roots, resulting in limited movement to other plant organs (Nagajyoti et al., 2010). Consequently, lower Cd concentrations compared to other metals are an expected pattern for many plant species (Alloway, 2013).

In conclusion, the mining site conditions significantly enhanced heavy metal accumulation in both *Hypericum* species; however, *H. scabrum* exhibited a higher accumulation capacity, whereas *H. lydium* showed a more limited but consistent increase. These findings indicate that the two species differ in their adaptive responses to mining ecosystem stress.

### 4.2 Translocation factor (TF) for the species across two different habitats

The comparison of translocation patterns between habitats and species revealed that TF values for most metals were higher in the mining site than in the non-mining area. Among the studied species, *H. lydium* exhibited a broader TF range, particularly for Fe and Mn. In contrast, Cr showed TF values below 1 in both species, indicating limited translocation, whereas Cd and Mn were characterized by TF values exceeding 1 in both species.

The higher TF values observed in the mining site suggest that both *Hypericum* species may enhance metal mobilization from roots to aerial tissues under increased metal exposure. The broader translocation capacity of *H. lydium*, particularly for Fe and Mn, indicates that this species may possess a greater ability to transport or tolerate these elements compared with *H. scabrum*. Conversely, the limited translocation of Cr in both species suggests a preferential retention strategy in root tissues, whereas the higher mobility of Cd and Mn may reflect their greater potential for internal transport within plant organs.

Previous studies have demonstrated that metal translocation capacity varies considerably among plant species and is influenced by both metal-specific characteristics and plant tolerance mechanisms. Translocation factor values greater than 1 are generally interpreted as an indication of efficient transfer of metals from roots to aboveground tissues, whereas values below 1 suggest restricted movement and predominant retention in roots (Raza et al., 2021). The regulation of metal transport between root and aerial organs is considered an important adaptive mechanism that allows plants to balance metal detoxification and tolerance under contaminated conditions (Sharma et al., 2023). Therefore, the higher TF values observed for Fe and Mn in *H. lydium* may reflect a greater internal transport capacity, while the low Cr translocation observed in both species may indicate stronger root immobilization of this element.

### 4.3 Comparative analysis of fatty acid composition in *Hypericum* species across different sites

The results of this study indicate that mining area conditions significantly alter the fatty acid composition in both *Hypericum* species, exerting a decisive influence on the balance between saturated and unsaturated fatty acids. The marked increase in total saturated fatty acids (ΣTSFA) in the mining area, contrasted with higher total unsaturated fatty acids (ΣTUSFA) in non-mining conditions, appears to be directly associated with heavy metal stress.

Heavy metal stress is known to induce oxidative imbalance through excessive production of reactive oxygen species (ROS), which can affect cellular membranes and trigger metabolic adjustments in plants. Previous studies have demonstrated that plants respond to metal-induced oxidative stress through several protective mechanisms, including antioxidant activation, metal sequestration, and modifications in cellular components to maintain physiological stability (Raza et al., 2021). Changes in membrane lipid composition are considered one of the adaptive responses that may contribute to the maintenance of membrane integrity under stress conditions. Therefore, the alterations in saturated and unsaturated fatty acid proportions observed in the present study may reflect a stress-induced adjustment in lipid metabolism associated with the tolerance responses of *H. scabrum* and *H. lydium* to mining-related heavy metal exposure.

Previous studies have shown that saturated fatty acids, especially palmitic (C16:0) and stearic (C18:0) acids, serve as key components of protective polymers such as cutin and suberin, which form physical barriers shielding the plant from heavy metals. These compounds protect the plant against water loss, mechanical damage, pathogens, and the entry of heavy metals into cells (Pollard et al., 2008; Beisson et al., 2012; Graça, 2015; Fich et al., 2016; He et al., 2018). In *H. scabrum*, the exceptionally high ΣTSFA levels observed in root tissues suggest that tissues in direct contact with metals activate a stronger protective response. Since roots are the first organs to encounter and accumulate heavy metals, the sharp increase in ΣTSFA in this organ likely represents an adaptive defense strategy. The observed increases in palmitic (C16:0) and stearic (C18:0) acids in the mining area support this overall trend. Although increases were observed in both species, the more pronounced changes in *H. scabrum* indicate species-specific physiological strategies for coping with metal-induced stress.

The low levels of unsaturated fatty acids (UFAs) in mining areas can be explained as follows. Mining sites are contaminated with heavy metals, such as iron, cadmium, copper, and chromium, which induce excessive production of reactive oxygen species (ROS) in plants (Saxena et al., 2016; Da Conceicao Gomes et al., 2017; Kawade et al., 2025). UFAs, particularly the 18-carbon species, are highly susceptible to ROS attack due to their double bonds (Guo et al., 2012). ROS target these polyunsaturated fatty acids in cellular membranes, initiating lipid peroxidation (Das and Roychoudhury, 2014; Kawade et al., 2025). This chemical attack leads to the degradation of UFAs. Therefore, in metal-stressed mining environments, UFA levels are reduced due to this “consumption” process (He and Ding, 2020; Kawade et al., 2025).

Unsaturated fatty acids, including oleic (18:1), linoleic (18:2), and α-linolenic (18:3) acids, act as critical “general defenders” in plant strategies against both biotic and abiotic stresses (He et al., 2018; He and Ding, 2020). In non-contaminated areas, where plants are not subjected to intense oxidative stress, UFAs are maintained at basal levels to serve as a preparatory defense against potential future biotic (pathogen, insect) or abiotic stresses (He and Ding, 2020). Consequently, in mining areas, heavy metals either degrade UFAs through peroxidation or limit their synthesis, resulting in lower levels. In contrast, in non-contaminated environments, the absence of such destructive pressures allows UFAs to be preserved at high levels, supporting plant growth and adaptive defense strategies. Comparison with the study by Shafaghat (2011) indicates substantial agreement, particularly with non-mining site samples. In both studies, palmitic, stearic, linoleic, and linolenic acids were identified as the predominant fatty acid components. Similarly, Özen and Başhan (2003) and Roumaissa et al. (2025) reported the dominance of linolenic, linoleic, and palmitic acids. These observations suggest that the fatty acid composition identified in the present study is largely consistent with the existing literature.

In summary, one of the earliest and most sensitive effects of heavy metal stress is observed in lipid metabolism. Unsaturated fatty acids (UFAs), due to their double bonds, are particularly susceptible to lipid peroxidation (Upchurch, 2008). Metal-induced oxidative stress targets UFAs in membrane phospholipids, reducing membrane fluidity and affecting cellular functions. This process is generally accompanied by a relative increase in total saturated fatty acids (ΣTSFA) and a decrease in total unsaturated fatty acids (ΣTUSFA) (Elloumi et al., 2014). The increase in saturated fatty acids is not merely a passive outcome but also represents an adaptive response. Saturated fatty acids such as palmitic (16:0) and stearic (18:0) contribute to cutin and suberin biosynthesis, forming hydrophobic barriers on the plant surface (Beisson et al., 2012; Fich et al., 2016). These structures not only limit water loss and pathogen entry but also restrict the diffusion of heavy metal ions into cells (Pollard et al., 2008; Graça, 2015). Therefore, the observed increase in ΣTSFA in *Hypericum* species exposed to metal stress in the mining area likely reflects an adaptive strategy to cope with the local environmental conditions.

### 4.4 Comparative palynological analysis of *Hypericum* species from contrasting sites

The obtained data indicate that the presence of mining-related environmental stress induces palynological alterations at varying magnitudes across species.

In *H. scabrum*, the variations in the polar and equatorial axes, as well as in the P/E ratio, were negligible, remaining approximately 1-fold. The absence of changes in pollen shape and size class indicates that this species exhibits greater morphological stability under the environmental stress conditions of the mining site. The approximately 1.10-fold increase in exine thickness, however, suggests a limited thickening of the protective wall structure.

In contrast, *H. lydium* displayed increases in the polar axis (1fold), P/E ratio (1fold), exine (1fold), and intine (1fold) under mining conditions, accompanied by a shift in pollen shape from prolate-spheroidal to subprolate. These changes indicate the development of a more pronounced morphometric response to environmental stress factors. Notably, the increase in the P/E ratio reflects a tendency toward a more elongated pollen morphology.

Previous studies have demonstrated that heavy metal contamination can negatively affect plant reproductive processes by disrupting cellular homeostasis, inducing oxidative stress, and altering developmental pathways associated with pollen formation and function (Keyster et al., 2020; Zhu et al., 2021). Heavy metal-induced accumulation of reactive oxygen species (ROS) may interfere with cellular processes during anther development, pollen maturation, and pollen tube growth, resulting in changes in pollen viability and morphological characteristics (Yousefi et al., 2011; Wang et al., 2015). However, the magnitude of these effects is not uniform among plant species and may depend on metal accumulation patterns, physiological tolerance mechanisms, and the ability of plants to regulate stress-induced cellular damage (Keyster et al., 2020; Zhu et al., 2021). Therefore, the contrasting pollen responses observed between *H. scabrum* and *H. lydium* in the present study may reflect species-specific differences in reproductive sensitivity and adaptive capacity under mining-associated heavy metal stress.

Overall, when an element-based evaluation was conducted to assess the relationship between elemental accumulation in floral tissues and palynological alterations, a clear association was identified between the elevated heavy metal load in flowers from the mining site and observed palynological changes. Despite increased metal accumulation in the floral tissues of *H. scabrum*, pollen morphology remained largely stable. In contrast, in *H. lydium*, the increased metal burden (particularly of Al, Fe, and Mn) in floral tissues was found to induce pronounced changes in both the P/E ratio and pollen shape.

It is well established that heavy metal accumulation in plants exerts significant effects not only on vegetative growth but also on reproductive biology. In particular, pollen development represents a highly sensitive process to environmental stress factors, and numerous studies have demonstrated that heavy metal stress can markedly affect pollen morphology, viability, and germination capacity (Wang et al., 2015; Muradoglu et al., 2017; Braga et al., 2023). In the present study, *H. scabrum* was found to accumulate notably high concentrations of Al, Fe, and Mn in floral tissues. Increased metal accumulation in generative tissues may lead to various morphological and structural alterations during pollen development. Heavy metal stress can influence the synthesis of sporopollenin, a key component involved in pollen wall formation, thereby causing modifications in pollen shape and exine structure (Parrotta et al., 2015). Therefore, the palynological differences observed in individuals growing in the mining area are likely to be a consequence of heavy metal-induced stress.

Heavy metals can also enhance the generation of reactive oxygen species (ROS), leading to cellular damage during pollen development. Oxidative stress occurring throughout this process may result in alterations in pollen wall thickness, the formation of abnormal pollen grains, and a reduction in pollen viability (Xie et al., 2022). In this context, the elevated accumulation of metals, particularly in floral tissues, can be considered a key factor explaining the variations observed in palynological parameters. Similarly, numerous studies have reported that environmental pollutants can exert pronounced effects on pollen morphology. For instance, in plants growing in regions characterized by intense urban and industrial pollution, changes have been observed in pollen size, shape, and exine ornamentation (Gill and Tuteja, 2010). These findings highlight that environmental stress factors can directly influence the reproductive biology of plants.

When the results of this study are evaluated collectively, it becomes evident that the increased metal accumulation in the mining area is translocated to floral tissues, and this condition may be associated with the observed alterations in palynological characteristics. Therefore, a direct causal relationship may exist between heavy metal accumulation and palynological changes, particularly manifested through generative tissues that are sensitive to metal-induced stress. This finding suggests that palynological traits in plants growing in mining ecosystems may serve as effective biological indicators of environmental stress.

### 4.5 Relationship between heavy metal accumulation, fatty acid composition, and pollen morphological responses

The observed correlation patterns suggest that Al, Cr, and Mn have stronger associations with fatty acid composition in both *Hypericum* species, whereas Cd exhibits a limited relationship with fatty acid content. These findings indicate that the influence of heavy metals on fatty acid composition may vary depending on the element type.

The t-test results reveal species-specific responses of pollen morphology to mining-related environmental stress. The limited changes observed in *H. scabrum*, mainly associated with the exine layer, suggest a relatively stable pollen structure under heavy metal exposure. In contrast, the broader alterations detected in *H. lydium*, involving both pollen dimensional traits and exine structure, indicate a higher degree of morphological sensitivity to mining-site conditions. These findings suggest that the impact of heavy metal stress on pollen morphology varies between species, with *H. scabrum* exhibiting a more constrained response and *H. lydium* showing a more pronounced morphological adjustment.

Previous studies investigating plant responses to heavy metal stress have generally focused on individual aspects, such as metal accumulation, oxidative stress regulation, or physiological tolerance mechanisms. Recent research has emphasized that plant adaptation to heavy metals involves complex interactions among metal uptake and transport, cellular defense responses, and metabolic adjustments rather than a single tolerance mechanism (Keyster et al., 2020; Sharma et al., 2022). Moreover, integrating multiple biological indicators has been suggested as a valuable approach for improving the understanding of plant responses under contaminated environments (Anjitha et al., 2023). In this context, the simultaneous evaluation of heavy metal accumulation, translocation patterns, fatty acid composition, and pollen morphological characteristics in *H. scabrum* and *H. lydium* provides additional information on species-specific responses to mining-associated stress conditions. The findings of the present study contribute to a better understanding of how different physiological and reproductive traits may vary between closely related species exposed to similar environmental pressures.

## 5 Conclusion

This study reveals that mining site conditions exert interrelated yet species-specific effects on heavy metal accumulation, palynological traits, and fatty acid composition in *Hypericum scabrum* and *Hypericum lydium*. Significant increases in all measured elements were observed at the mining site, with particularly notable metal accumulation exceeding 50-65-fold in certain organs of *H. scabrum*. Despite the elevated metal load, palynological data indicate that pollen morphology in *H. scabrum* remained largely stable. This finding was also supported by t-test results, which indicated a statistically significant difference (p < 0.05) only in exine thickness. In contrast, in *H. lydium*, increased accumulation of Al, Fe, and Mn in floral tissues led to pronounced changes in the P/E ratio, wall thickness, and pollen shape, resulting in a shift toward a more prolate morphology. These differences were confirmed by independent samples t-test results, which revealed statistically significant changes (p < 0.05) in polar axis, equatorial axis, and exine thickness in *H. lydium*. The observed alterations in pollen morphology, particularly variations in the P/E ratio, highlight the significant impact of heavy metal stress on reproductive structures and indicate species-specific differences in reproductive tolerance. At the biochemical level, the increase in total saturated fatty acids and the decrease in unsaturated fatty acids in the mining site suggest a protective adaptive strategy against heavy metal stress. This relationship was statistically supported by positive correlations of Al, Cr, and Mn with TSFA, and negative correlations with TUSFA. Moreover, TF values indicated that *H. lydium* exhibits a higher translocation capacity for Fe and Mn, whereas Cd showed low translocation capacity in both species. Overall, these findings demonstrate that mining ecosystems create a complex stress environment in *Hypericum* species, simultaneously affecting metal accumulation, reproductive biology, and fatty acid metabolism, and that the species develop distinct morphological and physiological adaptation strategies in response to these conditions.

In summary, plant responses to heavy metal stress in mining ecosystems are not limited to metal accumulation alone; they involve a multi-layered adaptive process encompassing reproductive biology and metabolic regulation. These results highlight that heavy metal stress triggers integrated morphological and metabolic adaptation responses in plants, providing important insights into understanding plant tolerance strategies in mining ecosystems.

## Data Availability

The original contributions presented in the study are included in the article/**Supplementary Material**. Further inquiries can be directed to the corresponding author.
